# Omics strategies for revealing *Yersinia pestis* virulence

**DOI:** 10.3389/fcimb.2012.00157

**Published:** 2012-12-13

**Authors:** Ruifu Yang, Zongmin Du, Yanping Han, Lei Zhou, Yajun Song, Dongsheng Zhou, Yujun Cui

**Affiliations:** Beijing Institute of Microbiology and EpidemiologyBeijing, China

**Keywords:** *Yersinia pestis*, omics, virulence, genomics, transomics

## Abstract

Omics has remarkably changed the way we investigate and understand life. Omics differs from traditional hypothesis-driven research because it is a discovery-driven approach. Mass datasets produced from omics-based studies require experts from different fields to reveal the salient features behind these data. In this review, we summarize omics-driven studies to reveal the virulence features of *Yersinia pestis* through genomics, trascriptomics, proteomics, interactomics, etc. These studies serve as foundations for further hypothesis-driven research and help us gain insight into *Y. pestis* pathogenesis.

Omics is a collective concept of high-throughput studies for understanding life (Morrison et al., 2006) using the integrative strategies of genomics, proteomics, transcriptomics, and metabolomics, as well as the newly developed omics strategies of RNomics (Bhattacharjee et al., 2012), lipidomics (Hartler et al., 2012), kinomics (Kindrachuk et al., 2012), glycomics (Turnbull and Sasisekharan, 2010), peptomics (Olsen et al., 2002), antigenomics (Levesque et al., 2012), chemomics (Wang et al., 2008), etc. A detailed discussion on omics can be found in the Wikipedia website (http://en.wikipedia.org/wiki/Omics). The development of high-throughput technologies often yields large datasets that require extensive bioinformatic integration to apply omics in biological research. Combining omics strategies to elucidate specific features of an organism has become a trend, providing a unique opportunity to gain holistic understanding of the physiological and pathological characterization of the studied organism. The word “trans-omics” is also used to describe this type of studies (Tuohy et al., 2009; Yang et al., 2011b; Ogata et al., 2012). A multitude of extensive reviews introducing different omics technologies exist (Morrison et al., 2006; Kolker, 2009; Holmes et al., 2010; Knox, 2010; Mahapatra, 2010; Ning and Lo, 2010; Wild, 2010), and we do not intend to repeatedly introduce these concepts and their related techniques in this review. We aim to provide a summary of the applications of omics strategies in studying *Yersinia pestis*, particularly in revealing its virulence.

## Genomics for uncovering *Y. pestis* virulence-associated genes

Some fragments or genes in *Y. pestis*, such as plasmids (pCD1, pMT1, and pPCP1) and chromosomal loci (*pgm* locus and pH6 antigen coding genes), have already been identified as virulence-associated even before its genome was decoded (Perry and Fetherston, 1997). The first genome of *Y. pestis* (strain CO92) offered unprecedented opportunities for understanding the virulence traits of this deadly pathogen (Parkhill et al., 2001). In addition, two other *Y. pestis* strain whole genomes, KIM and 91001, were decoded (Lindler et al., 1998; Perry et al., 1998; Deng et al., 2002; Song et al., 2004). These findings provided the opportunity to uncover virulence-associated genes due to the avirulent nature of 91001 in humans through comparative genomics (Zhou et al., 2004a).

### Virulence genes common in *Y. pestis* and *Y. pseudotuberculosis*

Although *Y. pestis* and *Y. pseudotuberculosis* are nearly identical at the genomic level, they cause very different diseases. Population genetics analysis revealed that *Y. pestis* evolved from *Y. pseudotuberculosis* no earlier than 26,000 years ago (Achtman et al., 1999; Morelli et al., 2010). As a recently emerged pathogen, *Y. pestis* shares some virulence determinants with its ancestor, *Y. pseudotuberculosis*, a gastrointestinal pathogen.

Type III secretion system (T3SS) is a well-known anti-host system responsible for the virulence of many pathogenic bacteria. T3SS in *Y. pestis* is composed of a secretion machinery, a set of translocation proteins, a control system, and six Yop effector proteins (Cornelis, 2002). *Y. pestis* injects effectors into the cytosol of eukaryotic cells when docking on the surface of host cells, thereby suppressing phagocytosis and host inflammatory response. T3SS in *Y. pestis* is encoded by a 70 kb plasmid, termed as pCD1, which is also found in two other pathogenic *Yersinia* species, *Y. pseudotuberculosis* and *Y. enterocolitica*. The loss of T3SS is sufficient to render *Y. pestis* completely avirulent, even when the bacteria are directly introduced into the bloodstream (Viboud and Bliska, 2005).

Escaping from macrophages at the early stage of infection is a vital step for *Y. pestis*, a facultatively intracellular pathogen. Interestingly, the ability to survive and replicate in macrophages is conserved in *Y. pestis* and *Y. pseudotuberculosis* (Pujol and Bliska, 2003). RipABC, MgtCB, Ugd, Yfe, and Feo have been shown to be required for the replication of *Y. pestis* in macrophages (Zhou and Yang, 2009).

Iron is well established as an essential nutrient chelated by mammalian proteins, lessening its availability to invading pathogens. Iron acquisition is critical to the survival of pathogenic bacteria during infection. Several iron acquisition systems have been characterized or annotated in *Y. pestis*, and at least two (Ybt and Yfe) of them have been proven essential to its full virulence (Gao et al., 2008; Sebbane et al., 2010). Ybt, also known as the high-pathogenicity island in the 102 kb *pgm* locus (Fetherston and Perry, 1994), is necessary for iron acquisition at the flea bite site and in the lymphatic system. On the other hand, Yfe is likely to exert effects in the later stages of the disease, i.e., blood-borne systemic dissemination (Gao et al., 2008).

### *Y. pestis*-specific virulence-associated genes

*Y. pestis* and *Y. pseudotuberculosis* have considerably similar genome contents. For instance, 75% of the annotated genes in the IP32953 chromosome of *Y. pseudotuberculosis* have no less than 97% identity (nucleotide level) to their homologs in *Y. pestis* (Chain et al., 2004). Horizontal gene transfer (HGT), also referred to as lateral gene transfer, is a major force in the evolutionary scenarios of bacteria (Gogarten et al., 2002). Some *Y. pestis*-specific genes acquired or lost by HGT are responsible for its unique virulence.

One major step in the speciation of *Y. pestis* is the acquisition of two unique virulence plasmids, pPCP1, and pMT1 (Achtman et al., 2004). Plasmid pPCP1 encodes the plasminogen activator (Pla), which is essential to both bubonic and primary pneumonic plagues (but not the primary and secondary septicemic forms) (Sodeinde and Goguen, 1988; Sodeinde et al., 1992), promotes *Y. pestis* dissemination from peripheral infection routes, and is responsible for the flea-borne transmission of the plague (Sebbane et al., 2006a; Lathem et al., 2007).

Plasmid pMT1 encodes an F1 antigen and murine toxin (Ymt). *Y. pestis* expresses a unique capsule-like F1 antigen at 37°C but not at 26°C, which are the human and flea body temperatures, respectively. F1 antigen provides *Y. pestis* the ability to block phagocytosis through a mechanism different from those of T3SS and pH 6 antigen (Du et al., 2002). Ymt shows phospholipase D activity but does not play a direct role in mouse plague infection (Hinnebusch et al., 2000). Further studies have revealed that the phospholipase D activity of Ymt is required for the survival of *Y. pestis* in the midgut of fleas by affording protection against a cytotoxic digestion product of blood plasma in the flea gut. By enabling the colonization of the flea midgut, Ymt acquisition may facilitate the transition of *Y. pestis* to an obligate flea-borne transmission style (Hinnebusch et al., 2002).

Various plasmids have been identified in different *Y. pestis* strains, such as 6-kb pYC (Dong et al., 2000), 22-kb pCRY (Song et al., 2004), and the 183 kb multiple antibiotic resistance plasmid pIP1202 (Welch et al., 2007). However, none of them have been proven to be involved in the virulence of *Y. pestis*.

A microarray-based genomic comparison of *Y. pestis* has identified an *Orientalis* strain-specific chromosomal region termed as DFR13 (Zhou et al., 2004b). This region encodes a filamentous prophage (YpfF) (Gonzalez et al., 2002), which is also called CUS-2(Cathelyn et al., 2007; Derbise et al., 2007). Deletion of YpfF from the *Y. pestis* genome does not affect its ability to colonize and block the flea proventriculus, but results in an alteration of *Y. pestis* pathogenicity in mice. Although YpfF is stably integrated into the genome of biovar *Orientalis* strains of *Y. pestis*, it forms an unstable episome in other *Y. pestis* biovars (Derbise et al., 2007). Screening YpfF in a larger collection of *Y. pestis* strains has confirmed this conclusion (Li et al., 2008a).

The identification of virulence genes other than the aforementioned ones from *Y. pestis*-specific genes is laborious and difficult. A detailed *in silico* comparative analysis of *Y. pseudotuberculosis* and *Y. pestis* has revealed only eight chromosomal loci (six regions and two genes) specific to *Y. pestis*. Signatures of integration by site-specific or homologous recombination are identified for most of them, suggesting they are imported by HGT. Intriguingly, depletion mutants of these loci exhibit no defect during growth *in vitro*. All depletants are also fully virulent and do not show detectable differences in the capacity to block the flea midgut. They also do not produce stable and persistent infection through subcutaneous or respiratory (aerosol) routes of infection. These results suggest that chromosomal acquisition may not have been of major importance in the remarkable change in life cycle that has accompanied the emergence of *Y. pestis* (Derbise et al., 2010).

### Genome decay

Genome decay (gene inactivation and gene loss), a common theme in the evolution of bacterial pathogens, occurs when a gene (or gene cluster) is no longer used by the microbe or when a microbe attempts to adapt to a new ecological niche (Cerdeno-Tarraga et al., 2004). Loss of the unnecessary genetic elements offers the pathogens better fitness when new niches are encountered.

A major finding in the first published *Y. pestis* genome is that CO92 harbors a large number of insertion sequences (IS) that comprise 3.7% of the genome, which is far more than in most other known bacteria. IS interruption and frameshift mutations have shaped 149 pseudogenes in the CO92 genome (Parkhill et al., 2001). A brief comparison of the CO92 and *Y. pseudotuberculosis* IP32953 genomes identified 317 genes absent from *Y. pestis*, which means that a large proportion of *Y. pseudotuberculosis* genes (13%) are no longer functional (inactivated or absent) in *Y. pestis* (Chain et al., 2004). The accumulation of pseudogenes may promote the speciation and microevolution of *Y. pestis* (Tong et al., 2005).

A comparison of the genomes of *Y. pestis* and *Y. pseudotuberculosis* has identified nine loci (five genomic regions and four individual genes), which are supposed to be present in *Y. pseudotuberculosis* and absent in *Y. pestis*. The deletion of R1, a region predicted to encode the methionine salvage pathway, altered the pathogenicity of mutant *Y. pseudotuberculosis*. Interestingly, R1 is present and conserved in two *Y. pestis* strains on the ancient lineage, implying R1 loss as an early step in *Y. pestis* microevolution. Region R3 has also been proven to be sequentially lost in the *Y. pestis* genomes (Pouillot et al., 2008). These gene-loss events reflect past decays of the *Y. pestis* genome, which is still actively evolving as an obligate intercellular pathogen with minimized genome (Moran, 2002; Ochman, 2005).

For example, *yadA* and *inv* genes respectively encode for the major adhesin and invasin in *Y. pseudotuberculosis*, which are essential for this enteropathogen to adhere onto host intestinal surfaces and invade epithelial cells (El Tahir and Skurnik, 2001; Superti et al., 2005). Both genes are inactivated in *Y. pestis* (Rosqvist et al., 1988; Simonet et al., 1996; Parkhill et al., 2001). Another important pseudogene in *Y. pestis* is *ureD*, which is inactivated by a premature stop codon, resulting in a malfunctioned *ure* operon (Sebbane et al., 2001). Urease is necessary for the oral transmission of *Y. pseudotuberculosis*; however, it is redundant in the flea-host-flea cycle of *Y. pestis* and may have been abandoned during its process of host adaption (Chen et al., 2010).

*Y. pestis* expresses rough LPS lacking the O antigen because of the inactivation of five genes in the O-antigen gene cluster (Parkhill et al., 2001). The loss of O-antigen and expression of rough LPS are believed to facilitate the Pla functions and invasiveness of *Y. pestis* (Kukkonen et al., 2004).

The transcriptional regulator RcsA is known to inhibit *Yersinia* biofilm formation. *rcsA* is functional in *Y. pseudotuberculosis* but is a pseudogene in *Y. pestis*. Substitution with the functional homolog from *Y. pseudotuberculosis* into *Y. pestis* abolishes its biofilm formation in fleas (Sun et al., 2008). The pseudogenization of *rcsA* is a clear case of positive selection during the evolution of *Y. pestis*.

### Metabolic enzymes and pathways

Ten years ago, Dr. Brubaker (1991) summarized the metabolic differences among *Y. pestis*, *Y. pseudotuberculosis*, and *Y. enterocolitca*, including the following metabolic defects in *Y. pestis*: biosyntheses of methionine, phenylalanine, threonine-glycine, and isoleucine-valine. The fermentation of rhamnose and melibiose is observed only in the biovar *Microtus* strains of *Y. pestis*, the closest lineage to *Y. pseudotuberculosis* that can ferment these two sugars (Zhou et al., 2004c; Zhou and Yang, 2009; Morelli et al., 2010). Both *Y. pestis* and *Y. pseudotuberculosis* cannot ferment sucrose and sorbitol compared with *Y. enterocolitica*, and all these three *Yersinia* species cannot ferment cellobiose (Brubaker, 1991). *Y. pestis* cannot produce aspartase (AspA), glucose-6-phosphate dehydrogenase, and urease; only *Y. enterocolitica* can synthesize ornithine decarboxylase; and only *Y. pestis* possesses constitutive glyoxylate bypass. In addition, among the three species, only *Y. pestis* is unable to assimilate low levels of NH_4_ at 26°C (Brubaker, 1991). The genome sequences of *Yersinia* are rapidly increasing; thus, a timely comparison of all metabolic differences among *Y. pestis* and *Y. pseudotuberculosis* can promote hypothesis-driven research on the evolution and pathogenesis of *Y. pestis*.

AspA catalyzes the deamination of L-aspartate to form fumarate, a component of tricarboxylic acid. Typical *Y. pestis* strains lack AspA activity, resulting in the excretion of L-aspartic acid at the expense of exogenous L-glutamic acid during expression of the low-calcium response. The accumulation of L-glutamate *in vivo* may radically alter the equilibrium of host amino acid pools, thereby contributing to enhanced lethality (Brubaker, 2007). Genomic analysis revealed a nonsynonymous substitution in codon 363 of *aspA* (valine to leucine) in typical *Y. pestis* isolates such as KIM and CO92 (Chain et al., 2004). Additional studies have revealed that this mutation (a replacement of the aliphatic amino acid leucine for another, valine, in AspA of *Y. pestis*) is not conservative. *K*_cat_/K_m_ of AspA-Leu363 in KIM is 500-fold lower than that of AspA-Val363 in *Y. pseudotuberculosis* (Viola et al., 2008). Screening AspA in more *Y. pestis* strains indicate that certain enzootic (pestoides) isolates of *Y. pestis* possess wild-type (WT) AspA. Additional variants of AspA (Phe363 and Ser363) are also found in other pestoides strains. All tested enzootic isolates produce biologically active AspA, although its specific activity exhibits significant variation depending on the codon 363 mutations (Bearden et al., 2009). The decreased activities of AspA in enzootic isolates may account for their attenuated virulence in mammals other than mice, and the functional AspA may serve as a biomarker for the avirulence of *Y. pestis* in humans (Bearden and Brubaker, 2010).

For a complete understanding of metabolic pathways and virulence, some works have been conducted based on bioinformatical, mathmatical, or experimental analyses (Navid and Almaas, 2009; Charusanti et al., 2011; Lv and Henderson, 2011; Navid, 2011), showing that *Y. pestis* genome-scale metabolic reconstruction can identify the metabolic weaknesses for a rational design of therapeutic agents. A combined metabolomic–genetic approach has demonstrated that virulence-associated secondary metabolite systems may shape bacterial primary metabolism independently of substrate consumption (Lv and Henderson, 2011).

As aforementioned, genomic analysis has revealed various mechanisms involved in the virulence of *Y. pestis*, including HGT, adaptive genome decay, and alterations in certain proteins and/or enzymes, which are far more complicated than expected. The influx of bacterial genome data have challenged the simplistic views that bacterial pathogens can be portrayed by identifying their virulence factors and that pathogens always evolve from non-pathogens by acquiring virulence genes by HGT. An “eco–evo” view of bacterial pathogenomics is very useful in understanding the evolution of complex virulence systems by taking the interaction of pathogens and hosts into account (Pallen and Wren, 2007).

## Transcriptomics for finding evidence of *Y. pestis* virulence

Bacteria are highly adaptive organisms that inhabit a broad range of ecological niches and constantly face variable environmental conditions. Thus, a pathogenic lifestyle requires strict control of both virulence gene expression and general stress responses. Comprehensive transcriptomics analysis benefits our understanding of the molecular determinants of bacterial pathogenesis and cellular regulatory circuits. These studies have refined the co-regulated genes, and have provided insight into the possible functions of uncharacterized genes and regulatory elements of *Y. pestis* (such as operons and DNA regulatory motifs). This kind of analysis provides an opportunity to gain a global view of the environmental modulation of gene expression patterns in *Y. pestis*, which is also very useful for the further genetic dissection of *Yersinia* pathogenicity.

### DNA microarray-based transcriptomics

The DNA microarray is an ideal tool for the genome-wide analysis of gene regulation at the transcriptional level. Comprehensive analysis of large sets of microarray expression data is useful for dissecting bacterial adaptation to various environments and for understanding bacterial gene transcriptional regulation (Goodman and Lory, 2004; Thompson et al., 2006). Environmental modulation of gene expression in *Y. pestis* is critical to its lifestyle and pathogenesis. To provide a comprehensive view of the environmental modulation of global gene expression in *Y. pestis*, we have analyzed the gene expression profiles of multiple stress conditions (Han et al., 2004, 2005a,b; Motin et al., 2004; Qiu et al., 2005, 2006; Zhou et al., 2005, 2006a,c), including temperature alteration, increased osmolarity, ion deficiency, antibiotic treatment, oxidative, and acidic stresses, antibacterial peptide treatment, as well as nutrition limitation.

#### Transcriptional regulation of virulence genes

The stress conditions used in array experiments are hypothesized to be encountered by *Y. pestis* during its infection and life cycle. Identification of the expression patterns of virulence genes within a wide range of environmental changes provides a reference for screening uncharacterized genes that show the same differential gene expression under the same stressful conditions.

The transmission and infection of *Y. pestis* can be roughly divided into distinct stages: maintenance in fleas, adhesion onto host surface, invasion into epithelial or endothelial cells, intracellular growth, antiphagocytosis, and extracellular proliferation. *Y. pestis* possesses a set of virulence determinants that promote infection in mammalian hosts and/or transmission by flea vectors. Different virulence genes have also been proven or proposed to be involved in different infection stages as reviewed (Perry and Fetherston, 1997; Zhou et al., 2006b). The array data supported the notion that *Y. pestis* has evolved the ability to regulate coordinately a large set of genes to survive an organized wide range of environmental perturbations (Han et al., 2007).

As previously described, the expression profiles of *Y. pestis* show that almost all the putative virulence genes of *Y. pestis* are differentially regulated upon temperature alteration (Han et al., 2004; Motin et al., 2004; Han et al., 2005b). Differential gene expression at these two temperatures is believed to allow the bacterium to colonize its host efficiently, leading to its pathogenesis. The known *Y. pestis* virulence genes also respond to many other environmental stresses. For example, the hemin storage locus *hmsHFRS* (Pendrak and Perry, 1991) is repressed by temperature upshift, high osmolarity, nutrition limitation, and streptomycin treatment. The *ymt* gene encoding *Yersinia* murine toxin (Lindler et al., 1998) is also regulated by temperature upshift and streptomycin treatment.

*Y. pestis* synthesizes several antiphagocytic factors, including F1 capsular antigen (Du et al., 2002), pH6 antigen (Huang and Lindler, 2004), and *Yersinia* outer proteins (Yops) (Cornelis et al., 1998). The expression of Yops is regulated by temperature alteration, increased osmolarity, and nutrition deficiency under normal Ca^2+^ conditions. These data suggest that the low-calcium response of T3SS appears to be triggered at the mRNA level by other environmental cues in addition to temperature upshift and Ca^2+^ limitation. F1 capsular antigen is expressed much more at 37°C than at 26°C (Simpson et al., 1990). pH6 antigen (PsaA), encoded by the chromosomal *psaA* gene, expresses *in vitro* between pH 5 and 6.7 from 35 to 41°C (Zav'yalov et al., 1996), or when bacteria live within phagocytic phagolysomes (Makoveichuk et al., 2003). The *psaEFABC* operon encodes a chaperone/usher pathway involved in the secretion and assembly of pH6 antigen as a polymer (fimbriae) on the surface of *Y. pestis* in macrophages (Zav'yalov et al., 1996; Payne et al., 1998). PsaE is believed to be a positive regulator of the *psaABC* locus and is required for the maximal expression of pH6 antigen (Lindler et al., 1990). A study showed that the *psaEFABC* locus is regulated by RovA (Cathelyn et al., 2006). The microarray data show that the F1 operon is upregulated upon temperature upshift, low pH, oxidative stress, low Mg^2+^, and nutrition deficiency. The *psaEFABC* locus is induced by temperature alteration, acid stress, low Mg^2+^, nutrition limitation, high salinity, and hyperosmotic stress. The synergistic operation of complicated microenvironments within mammalian hosts can be reasonably assumed to account for the full expression of these two loci (*psaABC* and *psaEFABC*).

To evaluate any specific contribution of one gene to host cell interaction, many efforts to measure the gene expression of *Y. pestis* systematically under relevant infection conditions have also been performed. *Y. pestis* transcriptomes *in vivo* have been analyzed in different infection models; different sets of differentially expressed genes represent distinct niches inhabited by *Y. pestis*. Two research groups have provided the *in vivo* transcriptome analysis of *Y. pestis* by establishing a primary pneumonic plague in mice (Lathem et al., 2005; Lawson et al., 2006). When compared with *in vitro* growth at 37°C, the plasmid pCD1-encoded T3SS and many genes located in the pigmentation (*pgm*) locus of the chromosome are significantly induced in the lungs of infected mice. Two other virulence determinants, Pla and pH6 antigen, are downregulated *in vivo* after 48 h of intranasal infection. In addition to the virulence determinants, genes involved in the detoxification of reactive oxygen species and multiple genes involved in the stress response are differentially regulated *in vivo*. The transcriptional profile of *Y. pestis* during the mouse bubonic plague shows similarities to and differences from that of *Y. pestis* during the primary pneumonic plague (Sebbane et al., 2006b). The *Y. pestis* T3SS and F1 capsule genes are highly expressed in both pneumonic and bubonic plague mouse models, whereas Pla is not regulated in the lymph node (bubonic plague). The upregulation of genes required for iron acquisition and for resistance to nitrosative stress suggests that iron deprivation and NO-induced stress are more severe in the rat bubo than in the mouse lung. Chauvaux et al. (2007) have analyzed the expression profile of *Y. pestis* CO92 incubated in decomplemented human plasma mimicking the septicaemic plague. Dozens of genes for iron acquisition or storage systems are particularly induced in the human plasma. pH6 antigen and four other fimbrial-like proteins encoding genes are also regulated *in vivo*. The ability of *Y. pestis* to survive inside macrophages has been established to be critical during the early stages of plague pathogenesis (Straley and Harmon, 1984; Charnetzky and Shuford, 1985). A number of stress-response genes, including those involved in the detoxification of reactive oxygen species, as well as several metabolic genes involved in macromolecule synthesis, are found strongly induced in intracellular *Y. pestis*. This finding is consistent with the presence of oxidative stress and nutrient starvation inside *Yersinia*-containing vacuoles (Fukuto et al., 2010). In the flea-borne transmission model, several genes involved in resistance to innate immunity (*phoP* and *mgtC*) and pathogenicity (*yadBC* and *Pla*) in mammals are found to be upregulated in the flea proventriculus (Vadyvaloo et al., 2010). The upregulation of these genes may enhance the survival and dissemination of *Y. pestis* in the early stage after transmission to the mammalian host.

#### Clustering analysis and functional classification of co-expressed genes

Clustering microarray expression data can be viewed as a data reduction process wherein observations of gene expression in each cluster can be over-represented. This process provides considerable insight into functional classes of co-expressed genes because the genes that are functionally related should be co-regulated and, consequently, should show similar expression profiles. Thus, clustering genes with similar expression patterns can potentially be utilized to predict the functions of gene products with unknown functions, as well as to identify sets of genes that are co-expressed and may play the same roles in different cell cycles (Eisen et al., 1998; Herrero and Dopazo, 2002). Clustering analysis of the entire microarray dataset was performed, and the four distinct clusters of co-expressed genes such as clusters I–IV are identified. The possible roles of uncharacterized genes may be inferred by referencing other members in each cluster. Cluster I consists of more than 70 genes, and most of which are functionally related to biosynthesis of ribosomal proteins. The ribosome is the site of protein synthesis and it determines the capacity of the cell to synthesize proteins, thereby determining the growth rate of bacteria. Most members of Cluster I are downregulated in response to a temperature shift from 26 to 37°C, high osmolarity, Mg^2+^ limitation, nutrition deficiency, and antibiotic treatment. Thus, *Y. pestis* appears to slow its growth rate under these conditions. Cluster II contains dozens of genes involved in iron/heme assimilation. Noticeably, almost all genes in this cluster are upregulated in response to iron scavenging in wild type (WT) strains, and to iron excess in ferric uptake regulator (*fur*) mutant grown at 26 or 37°C. Cluster III contains members of the *cy*s regulon, including *tauABCD*, *ssuEADCB*, *cysPUWAM*, and *sbp1*. These genes are regulated by most of the environmental stresses under study. Thus, sulfur metabolism may play important roles in the adaptation of *Y. pestis* to various environmental perturbations. Cluster IV includes *sdhCDAB* and *sucABCD* (involved in the tricarboxylic acid cycle), which have expression patterns similar to those of *nuoA-N* and *cyoABCDE* (involved in aerobic respiration). The microarray data show that these energy metabolism-related genes are downregulated upon heat shock, high osmolarity, Mg^2+^ limitation, and streptomycin treatment, but are upregulated upon chloramphenicol treatment. These results indicate that a general retardation of energy generation in *Y. pestis* may occur in response to such suboptimal growth conditions.

### cDNA library- or RNA sequencing (RNA-seq)-based transcriptional analysis of *Y. pestis* regulatory ncRNAs

Traditionally, studies on virulence-related regulations have been focused on the transcription factors that switch on or off relevant sets of genes in response to environmental cues. By contrast, the roles of small (noncoding) RNAs (sRNAs) in pathogenesis have only begun to be addressed. sRNAs are crucial regulators that enable the cell to modulate a wide range of physiological responses via various mechanisms. They are usually untranslated and 50–500 nucleotides in length. Most sRNAs interact with specific messenger RNAs (mRNAs) or protein targets by the trans-acting mode or structure-based interactions. The interaction results in the modulation of mRNA stability, translation, and protein activity (Narberhaus and Vogel, 2009; Waters and Storz, 2009). Hfq is postulated to be an RNA-binding protein, working in conjunction with many sRNAs. Interestingly, Hfq-mediated regulation seems to be implicated in the complete infective life cycle of the plague. Hfq is also shown to be a key regulator involved in *Y. pestis* stress resistance, intracellular survival, and pathogenesis (Geng et al., 2009), as well as a requirement for biofilm-mediated gut blockage in fleas by modulating the intracelullar levels of c-di-GMP (Bellows et al., 2012; Rempe et al., 2012). Hfq is now accepted to function by stabilizing and facilitating base-pairing between sRNAs and their cognate mRNAs (Vogel and Luisi, 2011). Therefore, Hfq acts by controlling the expression of many virulence-, biofilm-, and stress-associated genes, probably in conjunction with sRNAs. Attempts on the global screening of Hfq-dependent sRNAs have been made in *Escherichia coli* (Zhang et al., 2003). SgrS, an Hfq-dependent sRNA, is found to be induced under glucose-phosphate stress conditions and responsible for the destabilization of *ptsG* mRNA, which encodes the major glucose transporter of the phosphoenolpyruvate phosphotransferase system (Wadler and Vanderpool, 2007). SgrS is a dual-function sRNA with base-pairing and mRNA functions. A truncated SgrS homolog found in *Y. pestis* is still functional under glucose-phosphate stress (Horler and Vanderpool, 2009; Wadler and Vanderpool, 2009); however, its direct relevance with *Y. pestis* virulence is unknown. Hfq-dependent sRNAs reportedly contribute to *Y. pestis* virulence (Koo et al., 2011). Hence, the mechanism of known or newly discovered Hfq-dependent sRNAs involved in *Y. pestis* infection must be elucidated.

RNA-seq technology can enable the deep sequencing of cDNA generated from RNA preparations. Compared with tiling arrays, RNA-seq provides a better signal-to-noise ratio because of a reduced background and a higher dynamic range (Guell et al., 2011). Using this technology, 150 novel sRNAs have been recently identified in *Y. pesudotuboculosis*, the most closely related species to *Y. pestis*. One sRNA chosen for preliminary evaluation is found to play certain roles in the pneumonic plague (Koo et al., 2011). In a recently published study of our group, we used RNomics to find 43 highly abundant sRNAs in *Y. pestis* under multiple growth conditions (Qu et al., 2012). These highly expressed sRNAs under simulated conditions may play important roles in *Y. pestis* adaptability and pathogenesis. Analysis of the expression patterns of 29 candidate sRNAs shows that 24 sRNAs are highly abundant in *Y. pestis* upon entry into the stationary growth phase.

## Regulation of the expression of virulence determinants

The expression of virulence determinants, which allows *Y. pestis* to multiply on and within host cells and tissues, is strictly and coordinately regulated by various regulators. As a member of the MarR/SlyA family of transcriptional regulators that control the virulence of multiple bacterial pathogens (Ellison and Miller, 2006), RovA is required for the virulence of all three pathogenic *Yersinia* species (*Y. pestis*, *Y. pseudotuberculosis*, and *Y. enterocolitica*) through the regulation of various virulence loci (Revell and Miller, 2000; Nagel et al., 2001; Dube et al., 2003; Ellison et al., 2004; Cathelyn et al., 2006). In *Y. pseudotuberculosis* and *Y. enterocolitica*, RovA stimulates the transcription of *inv*, which encodes an invasin that mediates translocation across the intestinal epithelium (Revell and Miller, 2000; Nagel et al., 2001; Ellison et al., 2004; Heroven et al., 2004). The *rovA* null mutant of *Y. pestis* is much more attenuated after subcutaneous inoculation than after an intranasal or intraperitoneal route, which indicates the more important role of RovA in subcutaneous infection than in pneumonic or systemic ones (Cathelyn et al., 2006). In *Y. pestis*, RovA stimulates the transcription of the *psaEF*, *psaABC*, and CUS-2 prophage loci (Cathelyn et al., 2006). pH6 antigen encoded by *psaABC* acts as an antiphagocytic factor (Huang and Lindler, 2004) and plays a more important role in bubonic plague than in the pneumonic and septicemic forms, closely mimicking the role of RovA (Cathelyn et al., 2006). The RovA regulator still plays critical roles in the construction and functioning of the bacterial membrane, indicating the regulatory functions of RovA in antibiotic resistance and environmental adaptation (Yang et al., 2010).

PhoP and PhoQ constitute a classic regulatory two-component system (Groisman, 2001). The sensor protein PhoQ responds to low environmental Mg^2+^, acidic pH, and host-secreted antimicrobial peptides, and then phosphorylates the response regulator PhoP. As a transcription factor, phosphorylated PhoP either activates or represses its target genes by binding with their promoter-proximal DNA regions. A *phoP* null mutant of *Y. pestis* shows reduced ability to survive in macrophages and human neutrophils, as well as under *in vitro* conditions of low pH, oxidative stress, high osmolarity, and antimicrobial peptides (Oyston et al., 2000; Hitchen et al., 2002; O'Loughlin et al., 2010). This mutant is slightly attenuated in mice (Oyston et al., 2000); however, the LD_50_ of *Y. pestis* Δ*phoP* mutant does not differ from the WT strain for either the bubonic or pneumonic murine models of infection (Bozue et al., 2011). As a global regulator, PhoP controls a very complex regulatory cascade in *Y. pestis* (Li et al., 2008b; Perez and Groisman, 2009; Perez et al., 2009). The PhoP regulons in *Y. pestis* and *Salmonella enterica* considerably differ in terms of the functional changes in PhoP itself, as well as in the architecture of PhoP-dependent promoters. This difference allows the PhoP regulators to incorporate newly acquired genes into the ancestral regulatory circuits in these two bacteria (Perez and Groisman, 2009; Perez et al., 2009). The proven direct PhoP targets in *Y. pestis* include several genes that function in detoxification, protection against DNA damage, resistance to antimicrobial peptides, and adaptation to magnesium limitation (Li et al., 2008b). In particular, the *mgtCB* and *udg* loci that encode an Mg^2+^ transport system and a UDP-glucuronate decarboxylase for LPS modification, respectively, are required for the replication of *Y. pestis* in macrophages (Grabenstein et al., 2006). These PhoP-dependent mechanisms used by *Y. pestis* contribute to the intracellular growth of this pathogen.

The cyclic AMP receptor protein (CRP) controls the transcription of more than 100 bacterial genes/operons (Zheng et al., 2004). CRP is active only in the presence of cyclic AMP that behaves as a classic small-molecule inducer. The *crp* deletion leads to a huge attenuation of virulence of *Y. pestis* after subcutaneous infection of mice (Zhan et al., 2008). The expression of Pla, pesticin (Pst), and type III YOP secretion components depends on CRP in *Y. pestis* (Lee et al., 2007; Zhan et al., 2008, 2009). Specifically, CRP directly stimulates the expression of Pla (Kim et al., 2007; Lee et al., 2007; Zhan et al., 2008), a virulent factor essential to the bubonic and primary pneumonic plagues (Sodeinde et al., 1992; Lathem et al., 2007). Given that Pla specifically promotes *Y. pestis* dissemination from peripheral infection routes, the defective expression of Pla in the *crp* mutant significantly contributes to the huge loss of virulence of this mutant strain after subcutaneous infection (Zhan et al., 2008).

Fur is a predominant iron-regulating system in bacteria (Escolar et al., 1999). Fur directly controls almost all iron assimilation functions and a variety of genes involved in various non-iron functions, and thus governs a complex regulatory cascade in *Y. pestis* (Zhou and Yang, 2006; Gao et al., 2008). A variety of iron acquisition systems have been characterized in *Y. pestis*, and at least two (Ybt and Yfe) of them are proved to be required for full virulence (Bearden et al., 1998; Bearden and Perry, 1999). The *ybt* locus is transcribed into four operons (*fyuA*, *irp2*-*irp1*-*ybtUTE*, *ybtA*, and *ybtPQXS*) (Carniel, 1999, 2001). Fur repressed the whole *ybt* locus in response to excess extracellular iron. In addition to Fur, YbtA has been shown to be another transcriptional regulator that functions as an activator of *irp2*-*irp1*-*ybtUTE*, *ybtPQXS*, and *fyuA*, and as a repressor of its own transcription (Fetherston et al., 1996; Anisimov et al., 2005). Fur and YbtA share all four operons within the *ybt* locus as the direct targets at the transcriptional level (Gao et al., 2008).

The above regulators and their target virulence genes constitute a prototype of virulence gene regulatory network. Most of the abovementioned virulence factors are acquired through HGT. Thus, the newly acquired virulence genes, which are expressed at high levels during specific stages of infection, have been integrated into the host gene regulatory network controlled by the host regulators.

For many Gram-negative pathogens, hexa-acylated LPS can efficiently activate toll like receptor 4 (TLR4) signaling and further stimulate the host innate immune response. Both *Y. pestis* and *Y. pseudotuberculosis* do not carry the *lpxL* gene, which encodes an enzyme that transfers the secondary laurate chain to lipid A at 37°C and results in a tetra-acylated LPS with poor binding ability to TLR4, whereas *Y. enterocolitica* carries LpxL (HtrB) (Perez-Gutierrez et al., 2010). The remaining *lpxM* and *lpxP* can make a hexa-acylated lipid A in *Y. pestis* at ambient temperature, but at 37°C, plague bacteria produce mainly tetra-acylated LPS. Thus, a differential acylation of the lipid A in *Y. pestis* is a temperature-regulated process (Kawahara et al., 2002; Telepnev et al., 2009). Introducing *lpxL* into *Y. pestis* results in the formation of hexa-acylated lipid A at 37°C and ablating virulence in mice (Montminy et al., 2006). Clearly, the loss of *lpxL* plays a vital role in the immune evasion of *Y. pestis*, and an *lpxL* knock-in mutant of *Y. pestis* KIM strain has been proposed as a novel live vaccine against plagues (Sun et al., 2011).

## Host transcriptomic response to *Y. pestis* infection

DNA microarray technologies are widely used to investigate not only the bacterial transcriptome under various stimulant conditions, but also the host transcriptomic responses to microbial infections. *Y. pestis* infection-induced host transcriptomic responses have been investigated in both bubonic and pneumonic plague animal models, as well as in different types of cultivated cells. The combination of results from host transcriptomic responses, pathological studies on the tissues of infected animals, and pathogenic mechanisms of specific virulent factors of this pathogen has significantly improved our understanding of the crosstalk between *Y. pestis* and a host.

### *Ex vivo* transcriptional responses in infected cell cultures

*Y. pestis* can survive and replicate in macrophages by inhibiting the acidification of phagosomes *in vitro* and *in vivo* on bubonic mice model, but are killed in neutrophils (Lukaszewski et al., 2005). Evidence shows that neutrophils restrict the growth of *Y. pestis*, whereas macrophages do not (Lukaszewski et al., 2005). Macrophages actually provide a protected environment for organisms to synthesize their capsular layer and other anti-phagocytic mechanisms (Pujol et al., 2009). For a better understanding of the interactions between *Y. pestis* and a host at the early stage of infection, several groups have investigated the transcriptional response of cultivated macrophages or human peripheral blood lymphocytes to *Y. pestis* infection. Subrahmanyam et al. (2001) have applied a cDNA display technique to study mRNA level changes in human neutrophils following bacterial exposure using full virulence *Y. pestis* KIM5, pCD1-defective avirulent *Y. pestis* KIM6, and nonpathogenic *E. coli* K12 as the model bacteria. More than 300 genes are observed to be differently expressed in neutrophils exposed to bacteria, including genes of a variety of cytokines, receptors, apoptosis regulating products, and membrane trafficking regulators. A cluster of genes are *E. coli* and KIM6 responsive but unresponsive in neutrophils exposed to KIM5. For instance, the phagocyte oxidase system generating reactive oxygen is downregulated in KIM-infected neutrophils. By contrast, the free radical scavenging enzyme SOD2 is upregulated by nonpathogenic bacteria. Das et al. (2007) have reported transcriptomic response in cultured human monocytes and lymphocytes exposed to intracellular *Y. pestis*. Human monocytes are infected with *Y. pestis* bacteria grown at 26°C, and the extracellular bacteria are killed with gentamicin after 30 min. The ensuing transcriptional changes are largely due to the intercellular bacteria and reflect the host transcriptomic response at the intracellular life cycle of *Y. pestis*. Genes encoding cytokines as well as chemokines, transcription factors, inflammatory, and apoptosis-related genes are significantly changed, indicating that the infection of human monocytes with *Y. pestis* results in a strong inflammatory response (Das et al., 2007). Proinflammatory cytokines and chemokines such as TNF-α, macrophage inflammatory protein-1α (MIP-1α), MIP-1α, and interleukin-6 (IL-6) are upregulated 10–120-fold higher than untreated controls, whereas growth factor-β (TGF-β), MIP-2α, and placental growth factor are moderately upregulated (2–15-fold). The induction of the cytokine expression of TNF-α, IL-6, and MIP-1α peaks at 2–4 h and is eventually downregulated at 8 h, post-infection (p.i.) accompanied with the induction of the anti-inflammatory cytokine IL-10. This finding implies the inhibition of the initial inflammatory response. However, no significant transcriptional change can be detected in infected human lymphocytes, consistent with a previous report that *Y. pestis* preferentially infects monocytes (Marketon et al., 2005). The apoptosis of infected monocytes is inhibited by the downregulation of proapoptotic genes such as peripheral myelin protein 22 (PMP22), caspase-8, and complement component 5 receptor (C5AR), as well as the upregulation of apoptosis inhibitors such as B-cell lymphoma protein 2 (Bcl-2). This phenomenon is in contrast to the well-established function of YopJ, which has been shown to induce apoptosis in macrophages by blocking mitogen-activated kinase and NF-κB signaling events (Lemaitre et al., 2006; Mukherjee et al., 2006). This contradiction is probably due to the fact that Yops protein cannot be efficiently delivered into the host cells in an intercellular infection experiment. The cellular functions of homeostasis and coagulation are also persistently downregulated after exposure to *Y. pestis*, which favor clot formation and contribute to disseminated intravascular coagulation, a major cause of death from the plague.

To determine the contributions of Yops and T3SS to its pathogenesis, the transcriptional responses of J774A.1 or bone marrow-derived macrophages (BMDM) from BALB/c to an infection with *Y. enterocolitica* and various mutants with pYV defections (pYV-cured strain and Yop mutants including *yopJ*, *yopM*, and *yopH* deletions) are analyzed (Hoffmann et al., 2004). The results show that pYV-cured *Y. enterocolitica* induces a general inflammatory response in infected J774A.1 macrophages, whereas a WT *Y. enterocolitica* strain induces the expression of genes that have silencing functions in inflammatory responses and can successfully suppress this response. Similar results have been reported in the lymph node from a bubonic plague model, which shows significantly delayed immune response in WT *Y. pestis* but not in pYV^−^ mutant (Comer et al., 2010). A comparison of the transcriptional profiles induced by WT *Y. enterocolitica* and Yops mutants reveals that YopP (YopJ in *Y. pestis*) mediates the suppression of the inflammatory response; however, neither YopH nor YopM modifies the expression profile on macrophage genes.

A transcriptional response of BMDM from resistant C57BL/6 or susceptible BALB/c mice to infection with *Y. enterocolitica* has been reported. A few genes that activate NK cells, possess antibacterial properties, or are involved in sensing chemokines and LPS are more strongly induced by C57BL/6 BMDM in response to *Yersinia* sp. infection than BALB/c BMDM (Van Erp et al., 2006). These results indicate that although the host resistance factors modulate a very small portion of transcriptome, the expression of this cluster of genes affects the outcome of the disease.

### Transcriptional responses to the bubonic and pneumonic plagues

Studies using both bubonic and primary pneumonic plague animal models have revealed that host infections with *Y. pestis* show a remarkable biphasic feature in which the infection begins with an early anti-inflammatory phase followed by a proinflammatory phase (Nakajima and Brubaker, 1993; Sebbane et al., 2005; Bergsbaken and Cookson, 2009; Comer et al., 2010). In the bubonic plague, *Y. pestis* rapidly multiplies in draining lymph nodes at the early stage (6–36 h p.i.) with no detectable inflammation. The bacteria then rapidly replicate and disseminate in the blood to colonize the liver, spleen, and lungs. Host immune responses, including phagocyte infiltration, inflammatory cytokine production, and tissue necrosis appears only after 36 h p.i (Sebbane et al., 2005). Lathem et al. (2005) have also found this biphasic feature in a mouse model of primary pneumonic plague by histopathology inspection. Their results indicate that infection begins with an anti-inflammatory state in the first 24–36 h, and then rapidly progresses to a highly proinflammatory state by 48 h and death by 3 days. Similarly, robust neutrophil recruitment to the lungs is not observed until 48 h p.i. in a mouse pneumonic model by Liu et al. (2009). Proinflammatory chemokines are also undetected in bronchoalveolar lavage fluids during this period of infection.

#### Transcriptional responses to bubonic plague

The first transcriptomics study on the host response to *Y. pestis* infection was performed in spleens from intraperitoneally (i.p.) infected mice (Rogers et al., 2007). About 48 h after the mice were i.p. infected with 8 or 257 CFU of *Y. pestis* CO92, the expression of 534 genes were significantly modified in the high-dose infected group, with 384 genes downregulated and 150 genes upregulated. No significant gene expression change was detected in the low-dose infected group, which exhibited no mortality either. Thus, the host immune system successfully controlled the initial spread of *Y. pestis* when the challenge dose was sufficiently low. The altered genes primarily encode proteins in biological processes concerned with immune, cytoskeletal, and cell cycle functions, apoptosis, as well as protein degradation. The upregulated genes associated with immune functions include Src-like adapter, IL-18, TLR8, CD14, and dual specificity phosphatase 1. The downregulated genes contribute to the inhibition of immune response that involves B cell receptor/co-receptor signaling and subsequent B cell activation, including spleen tyrosine kinase, CD19, complement receptor 2, and E26 avian leukemia oncogene 1. The expressions of nearly 40 genes related to cytoskeletal remodeling are changed by a *Y. pestis* infection. This finding suggests that Yop effectors (including YopH, YopE, YopT, and YpkA), which can directly target small GTPase family members, exert their virulence function and paralyze the phagocytosis function of host cells.

Comer et al. (2010) have investigated the host response to *Y. pestis* in the lymph nodes of a rat bubonic model. Results show that transcriptomic response in lymph nodes can only be detected after the infection has progressed to septicemic plague (60 h p.i.), which includes the upregulation of some cytokines, chemokines, and other immune response genes. These results are in accordance with the previously reported biphasic features of the plague (Lathem et al., 2005). By contrast, the mutant of *Y. pestis* lacking the pCD1 plasmid invokes a significant transcriptome response at 36 h. The transcriptomic results are further confirmed by the pathologic observation that the lymph node of an animal infected with pCD1^−^ *Y. pestis* is characterized by sustained recruitment of increasing numbers of neutrophils and the successful clearance of bacteria. This feature highly differs from the WT *Y. pestis*-infected lymph node. These results show that the pCD1-encoded molecules may induce active immunosuppression at the early stage of the bubonic plague, as supported by a recently published work (Price et al., 2012). Price et al. (2012) have shown that *Y. pestis* rapidly creates a localized, dominant anti-inflammatory state that allows the survival and rapid growth of normally avirulent organisms; however, its progenitor *Y. pseudotuberculosis* has no such capability. Evidence shows that the T3SS effectors of enteric *Yersiniae* and *Y. pestis* are not functionally equivalent, or that other virulence factors specific to *Y. pestis* play a role (Balada-Llasat and Mecsas, 2006). Coinfection of WT and pCD1^−^ *Y. pestis* reportedly results in a similar transcriptional response at 36 h to that aroused by pCD1^−^ *Y. pestis* alone. Thus, WT *Y. pestis* cannot prevent the immune response aroused by pCD1^−^ *Y. pestis*. Nevertheless, some immune response genes show significant differences between rat infected with pCD1^−^ *Y. pestis* alone and that coinfected with WT *Y. pestis*. These results imply that a specific part of the immune system can be actively suppressed by pCD1-encoded T3SS (Comer et al., 2010). The IL-17 levels in the lymph node and the transcription of the gene for IL-17F are both significantly increased in response to *Y. pestis* infection (Comer et al., 2010). A recent report demonstrates that the increased level of IL-17 in the lungs of challenged B cell-deficient mice can improve survival (Lin et al., 2011), suggesting that IL-17-mediated cell immunity plays some roles in host defense against the pneumonic plague.

#### Transcriptional responses to the pneumonic plague

Two independent groups have reported the transcriptional responses of different mouse organs (including lungs, spleen, and liver) in an intranasally challenged mouse model of the pneumonic plague (Galindo et al., 2009; Liu et al., 2009). Both studies show a significantly higher number of differentially expressed genes in the lungs at 48 h than at 12 h p.i. The small number of changed genes in the lungs from animals exposed to *Y. pestis* is not accompanied by a significant increase in PMNs. Pathological change is also found during the early stage of infection, and strong cytokine production is not detectable until 48 h p.i. (Liu et al., 2009). CSF-3, MIP-3α, GRO-β, and IL-β are among the most commonly induced cytokines in the lungs at 48 h p.i. Both CSF-3 and GRO-β target the neutrophils to promote their differentiation, proliferation, and eventual recruitment to the sites of infection. The strong induction of these cytokines reflects a strong immune response at the later stages of the plague. Although significant discrepancies are observed among the mice, cDNA microarray, and data analysis used by the two groups, the majority of the alerted genes well coincided with one another based on a comparison of the published datasets (Galindo et al., 2009).

### Prospects and remaining questions

*Y. pestis* is considered to be a clone that branches from its progenitor *Y. pseudotuberculosis* (Achtman et al., 1999, 2004); however, it is highly virulent compared with its enteropathogenic ancestor. The two species share more than 90% DNA homology and a pCD1/pYV virulence plasmid. *Y. pestis* has acquired two additional plasmids, namely, pPCP1, and pMT1, facilitating its transmission by fleabite among additional functions. An interesting phenomenon is that the delayed immune response is diminished in a pCD^−^-*Y. pestis* infected bubonic rat model, in which a general inflammatory response is evoked in the infected bubo. This finding suggests that T3SS encoded by pCD1/pYV plasmid is responsible for the immune repression induced by *Y. pestis* (Comer et al., 2010). However, no difference is detected in mice subcutaneously challenged with *Y. pestis* or *Y. pseudotuberculosis* during the progression of the infection to the draining lymph nodes (before 2 days of infection) (Guinet et al., 2008). The subsequent *Y. pseudotuberculosis* infection induces massive PMN influx and the bacterial replication is contained, whereas PMN infiltration is absent in the *Y. pestis*-infected draining lymph nodes, which are typified by an invasion of the tissue by free-floating bacteria. This observation indicates that *Y. pseudotuberculosis*, harboring a pYV plasmid, cannot suppress the host defense response as its descendant, suggesting that chromosomally encoded virulence-associated factors play roles in this immune suppression. More detailed experiments on host transcriptomics can be designed to investigate the evolution of *Y. pestis* from a moderately virulent enteropathogenic to a highly virulent species.

Next-generation sequencing (NGS) technologies can simultaneously sequence millions of DNA molecules with high throughput efficacy at a lower cost (Wang et al., 2009; Wilhelm and Landry, 2009). NGS-based RNA-seq technologies directly determine the cDNA sequence and generate digital signals that provide highly quantitative and reliable measurements of transcriptional levels (Marioni et al., 2008; Mortazavi et al., 2008). These technologies have been proven to be a powerful alternative to DNA-microarray-based technology in transcriptomic studies, and can certainly affect this area. RNA-seq is particularly attractive for the quantitative detection of low-abundance transcripts with very high sensitivity, and for the unbiased detection of almost all kinds of transcripts in a given cell type, which is impossible for DNA microarray technology. The application of RNA-seq technology to *in vivo* and *in vitro Y. pestis* infection models can enable the collection of delicate and complicated data on host transcriptomic responses.

## Proteomics for studying *Y. pestis* virulence

Given the numerous genomic and transcriptomic analyses of *Y. pestis*, further research at the protein level (proteomics) can provide more information on the virulence of the pathogen.

Two-dimensional electrophoresis (2-DE) is extensively used as a conventional tool for exploring the virulence factor and the regulatory network involved in the pathogenicity of *Y. pestis*. Pieper et al. (2008) have characterized the periplasmic proteome of *Y. pestis* strain KIM6+ using a differential 2-DE display of proteins isolated from several subcellular fractions. They have found that several periplasmic proteins with unknown functions may play important roles in the *Y. pestis* life cycle. They have also compared the abundance changes at 26 and 37°C by analyzing subcellular proteomes, and found that many outer-membrane (OM) proteins, including cell adhesion protein Ail (y1324) and three putative small beta-barrel OM proteins (OMPs) (y1795, y2167, and y4083), are strongly increased at 37°C. By contrast, the Ail/Lom family protein y1682 (OmpX) is strongly increased at 26°C (Pieper et al., 2009). Some type VI secretion system proteins are only identified in membrane fractions of stationary-phase cells grown at 26°C. Chromy et al. (2005) have used the Ettan 2-D DIGE system to compare the protein expression of *Y. pestis* at both 26 and 37°C with and without 4 mM calcium. They have found the differential expression of several virulence-associated factors [including catalase-peroxidase (KatY), murine toxin (Ymt), Pla, and F1 capsule antigen (Caf1)], as well as several putative virulence factors and membrane-bound and metabolic proteins. These factors may represent new virulence determinants. Pieper et al. (2010) have compared proteomic changes in three *Y. pestis* (strain KIM6+) subcellular fractions (soluble periplasmic, cytoplasmic, and mixed membrane fractions) under iron deficiency at two physiologically relevant temperatures (26 and 37°C). They have found that five characterized *Y. pestis* iron/siderophore acquisition systems (Ybt, Yfe, Yfu, Yiu, and Hmu) and a putative iron/chelate OM receptor (Y0850) are increased in abundance in iron-starved cells. This result may contribute to the understanding of the important regulatory role of Fur. The iron–sulfur cluster assembly system Suf is functional in *Y. pestis* under iron-limiting conditions. All these comparative studies reveal numerous clues for further functional research on *Y. pestis*; however, the low coverage (usually 20–30% of all predicted proteins) of 2-DE restricts further analysis. Therefore, different technologies are used in some research. In our laboratory, we have developed a protein microarray consisting of virulence-associated proteins of *Y. pestis* to compare antibody profiles elicited by the WT and quorum-sensing (QS) mutant strain of this bacterium and to define the immunogens affected by QS. The results demonstrate that QS affects the expression of many virulence-associated proteins of *Y. pestis*, including F1, LcrV, KatY, and pH6 antigen (Chen et al., 2006). We have also used this protein microarray to study the host antibody responses to uncover seven *Y. pestis* proteins specifically expressed *in vivo* during infection (Li et al., 2011). Hixson et al. (2006) used the accurate mass and time (AMT) tag mass spectrometry (MS) method and clustering analysis to compare the abundance change in 992 *Y. pestis* proteins under four contrasting growth conditions (26 and 37°C, with or without Ca^2+^) that mimicked growth states in either a flea vector or mammalian host. They have identified unique biomarkers specifically related to growth conditions. The OMPs are analyzed for a nonpathogenic *Y. pestis* A1122 strain by liquid chromatography–tandem MS (Jabbour et al., 2010) because OMPs are often associated with virulence in Gram-negative pathogens. However, this study only compares the OMPs between *Y. pestis* and *E. coli*, and comparative proteome analysis between pathogenic and non-pathogenic *Y. pestis* can help identify virulence-associated proteins more efficiently.

The recent developments in multidimensional chromatography separation and advanced MS techniques considerably promote research on large-scale proteome profiling (Wall et al., 2000; Washburn et al., 2001; Peng et al., 2003; De Godoy et al., 2006). The implementation of high-profile MS instruments, particularly Fourier transform ion cyclone resonance MS and the novel Orbitrap MS, enables the identification of protein mixtures with high throughput and quality (Olsen et al., 2005; Chapman et al., 2006; Heurlier et al., 2006; Zubarev and Mann, 2007). In a typical research on the metal-reducing microorganism *Geobacter sulfurreducens* using ultra-high-pressure liquid chromatography and MS-based AMT strategy as well as 2-DE, the authors approach about 90% of the total predicted gene products (Riding et al., 2008). To understand better the physiology and pathogenesis of *Y. pestis*, we have carried out an in-depth proteomic analysis of *Y. pestis* strain 91001 at 26°C in a chemically defined medium that mimicks growth states in a flea vector. We have used an advanced LTQ-FT mass spectrometer equipped with a nanospray ion source and an Agilent 1100 Series binary high-performance liquid chromatography system (Zhou et al., 2012). The results demonstrate high coverage [a total of 1926 proteins (13,082 peptides) identified, accounting for 46.50% (1926/4142) of *Y. pestis* 91001] and high quality [less than 1% false discovery rate] using this combined strategy. The subsequent transcriptome analysis based on a whole genome DNA microarray of *Y. pestis* defines 1655 genes with 56.65% coincidence to the proteomic results. Through the comprehensive analysis of the activity of virulent factors involved in the entire life cycle of *Y. pestis* under *in vitro* flea-simulated conditions, the Hms system and murine toxin (virulence factors involved in *Y. pestis* maintenance in flea) are found to show high expression in our analysis. Some virulence factors are also activated to different extents, such as Pla involved in the adhesion and invasion in mammalian hosts, PhoP/PhoQ two-component system involved in intracellular growth, T3SS, iron acquisition systems (Ybt, Yfe, and Yfu), and Fur involved in extracellular growth and systemic infections. *Y. pestis* clearly utilizes a variety of survival strategies before invading its hosts.

Proteomics approaches have also been used to study the host–*Yersinia* interactions (Zhang et al., 2005a). Human monocyte U937 cells exposed to *Y. pestis*, *Y. enterocolitica*, and *Y. pseudotuberculosis* were analyzed by 2DE MS to reveal the host response at the proteomic level (Chromy et al., 2004). Several differentially expressed host proteins involved in protein synthesis, cytoskeletal interactions, immune responses, and apoptosis were identified, suggesting that a specific host protein response profile may be useful for diagnosis of disease without any information of possible pathogens. The same group also quantitatively reveals the protein expression differences from cytoplasmic, nuclear, and membrane fractions of host cells U937 by 2DE DIGE between *Y. pestis* and *Y. pseudotuberculosis* challenges (Zhang et al., 2005b). The functions of the differentially expressed proteins can provide insight into the different virulence and pathogenic mechanisms of these two genetically related pathogens.

Ponnusamy et al. (2011) have compared the protein expression differences between intracellularly and extracellularly grown *Y. pestis* using 2DE-MS strategy. The comparison reveals nine proteins that may be involved in stress responses to benefit the intracellular growth of the pathogen in mouse macrophages, including superoxide dismutase-A, inorganic pyrophosphatase, GrcA, DnaK, GsrA, H-NS, UreA, TerD, and TerE.

Our group have used a protein microarray and an enzyme-linked immunosorbent spot assay to evaluate both humoral and cellular immune responses to *Y. pestis* infection in long-term recovered plague patients (Li et al., 2012), laying a foundation for new diagnostic development and vaccine design.

## Interactomics for revealing *Y. pestis* virulence

Complicated interactions between the host and pathogen ultimately determine the outcome of a disease. The high-throughput yeast two hybrids (Y2H) system and affinity purification coupled with MS techniques have rapidly progressed in recent years. Accordingly, large-scale investigations on protein–protein interactions (PPIs) by a high-throughput strategy are now widely adopted to investigate the pathogen–host interactions. Despite these advances, we are far from the well understanding of pathogen–host interactomics. The successively published large-scale PPIs networks of human and model organisms provide valuable references for investigating protein interaction networks between pathogens and their hosts (Li et al., 2004; Lacount et al., 2005; Rual et al., 2005; Stelzl et al., 2005; Parrish et al., 2007). Viruses are more intensively studied in this aspect than bacterial pathogens partly because of the fact that viruses generally contain relatively small genomes. They also replicate inside the host cells, utilizing the host resources; thus, most of the viral proteins can interact with multiple interaction partners to succeed in this mission. Published virus–host infection networks include hepatitis C virus, Epstein–Barr virus, and Kaposi sarcoma-associated herpes virus (Uetz et al., 2006; Calderwood et al., 2007; De Chassey et al., 2008). More recently, the interaction networks between human and bacterial pathogens of *Y. pestis*, *Bacillus anthracis*, and *Francisella tularensis* have been reported (Dyer et al., 2010). These reports reveal that pathogens tend to interact with hubs (proteins interacting with a large number of partners) and bottlenecks (proteins connecting with many functional modules) in the human PPI network. Distinct pathogens also preferentially interfere with a different spectrum of cellular pathways to facilitate infection and dissemination.

In the study of Yang et al. (2011a), a total of 153 potential virulence-associated proteins of *Y. pestis* are chosen as baits to screen against the human spleen cDNA library using a direct Y2H strategy. More than 200 PPIs between *Y. pestis* and human proteins were identified, and a *Y. pestis*–human PPI network was constructed in combination with the published interactions obtained from literature. Results showed that *Y. pestis* is highly prone to interact with hub and bottleneck proteins essential to normal cellular functions, further supporting previous results that pathogens usually target the central proteins in the human PPI network (Dyer et al., 2008, 2010). The Kyoto Encyclopedia of Genes and Genomes (Aoki-Kinoshita and Kanehisa, 2007) pathways enriched in human proteins targeted by *Y. pestis* proteins highlight a number of important pathways in immune response, including the TLR and MAPK signaling pathways, leukocyte transendoepithelial migrations, focal adhesion, and cytoskeletal regulation. Interference with these pathways by *Y. pestis* helps create a benefit local environment for its replication by modulating the recruitment of leukocytes, inhibiting the phagocytosis of the host phagocytes and interrupting immune signaling events.

Three interaction networks between human and bacterial pathogens, including *Y. pestis*, have been reported using a random Y2H approach (Dyer et al., 2010). The human–*Y. pestis* interaction network reported in Dyer's work contains more than 4000 interactions between 1218 *Y. pestis* proteins and 2108 human proteins, which is a much larger dataset than that of Yang's study (Yang et al., 2011a). Only 22 of *Yersinia* bait proteins are shared and no common interaction exists for these 22 baits in the two datasets, probably and partly due to the fact that different approaches are adopted in the two studies (random Y2H vs. direct Y2H). In addition to the PPIs revealed by the high-throughput techniques described above, some methods have been developed to predict the interactions between host and microbial pathogens. Over 28,000 interactions between *Y. pestis* and human have been predicted. Some of these interactions can be highly relevant with the pathogenesis of the pathogen and are worthy of further investigation (Krishnadev and Srinivasan, 2011).

## Perspectives

Comprehensive trans-omic analyses, including genomic, transcriptomic, proteomic, and phenomic data, on *E. coli* have shown a typical example of a holistic understanding of its cellular physiology and metabolism (Yoon et al., 2012). However, the study is still quantitative and descriptive. Obtaining quantitative and dynamic data, as well as verifying in a large-scale the functions of the differences, is still a long-term task remaining to be tackled.

Most omics reports to date are mainly *in vitro* studies, and performing *in vivo* omics studies (such as on pathogen–host interactome in real-time monitoring) is still a challenge in terms of technical development. DNA sequencing and MS technologies prompt us to perform systems studies on pathogenic bacteria, including *Y. pestis*, and help us refine genome annotation (Payne et al., 2010; Schrimpe-Rutledge et al., 2012). As shown above, we have acquired large-scale data for its genomic, transcriptomic, sRNA, and proteomic information; however, we still cannot systematically integrate all these data because the experimental conditions for obtaining these data are not uniformly designed. These fragmented data provide us numerous clues for further studies, and sometimes we cannot decide which targets to choose when faced with many choices. We need to choose the highly and less virulent strains to humans when performing dynamic comparative omics studies, even when comparing with *Y. pseudotuberculosis*, to facilitate a systematic discovery of virulence-associated factors, regulation differences, and host response variations. These data are extremely critical for designing pharmaceuticals, developing diagnostics, and making plague countermeasures.

Schrimpe-Rutledge et al. (2012) have shown a good example of omics-driven genome annotation refinement using transcriptomic and proteomic analyses by comparing the epidemic strain *Y. pestis* CO92, non-epidemic *Y. pestis* strain Pestoides F, and *Y. pseudotuberculosis* PB1. Recently, Ansong et al. gave us another good example of comparative omics study by using genomics, transcriptomics, proteomics and metabolomics to elucidate *Yersinia* virulence mechanism (Ansong et al., 2012). They not only predicted a network of putative virulence factors consisting of 151 cluster members, including 34 pCD1-encoded proteins, 53 pMT1-encoded ones and 64 chromosomally encoded ones; but also revealed potential virulence roles of 11 pMT1-encoded genes (YPMT1.38c, YPMT1.39c, YPMT1.40c, YPMT1.41c, YPMT1.46Ac, YPMT1.49Ac, YPMT1.49c, YPMT1.52c, YPMT1.55c, YPMT1.88, and YPMT1.89) for larger animals, including humans.

To understand *Y. pestis* virulence using omics in future studies, careful design of cell or animal models and methods of correlating the results to human clinical data are needed. The association of a microbiome and its dynamics in gut or bronchia with plague development is also a considerable challenge to understand the plague from a different angle. To integrate all available omics data from fragmented studies on *Y. pestis*, we need to reannotate the *Y. pestis* genome meticulously, reconstruct its metabolic pathways (Navid and Almaas, 2009), and then integrate all available information for a systems understanding of its life, strengthening the growing importance of bioinformatics and bioinformaticists in the growing datasets provided by Omics research.

### Conflict of interest statement

The authors declare that the research was conducted in the absence of any commercial or financial relationships that could be construed as a potential conflict of interest.
